# An Evaluation of Substance Abuse Treatment and HIV Education on Safe Sex Practices in Cocaine Dependent Individuals

**DOI:** 10.1155/2014/912863

**Published:** 2014-03-04

**Authors:** Theresa M. Winhusen, Eugene C. Somoza, Daniel F. Lewis, Frankie Kropp, Jeff Theobald, Ahmed Elkashef

**Affiliations:** ^1^Addiction Sciences Division, Department of Psychiatry and Behavioral Neuroscience, University of Cincinnati College of Medicine, 3131 Harvey Avenue, Cincinnati, OH 45229, USA; ^2^Veterans Affairs Medical Center (VISN 10), 3200 Vine Street, Cincinnati, OH 45220, USA; ^3^National Rehabilitation Center, P.O. Box 55001, Abu Dhabi, United Arab Emirates

## Abstract

*Background.* There is a strong association between crack/cocaine use and increased sexual risk behavior, but little research on the efficacy of HIV education for decreasing such behavior in crack/cocaine-addicted individuals in substance abuse treatment. *Method.* Datasets from two cocaine dependence trials including either one or three HIV education sessions, respectively, were analyzed for changes over time in the proportion of participants practicing safe sex. A pooled dataset from two earlier trials not offering HIV education was also analyzed. *Results.* We included 83 participants from the 1-session trial and 65 participants from the 3-session trial. Both sets of participants evidenced a significant increase in the proportion of participants having safe sex with casual partners. Participants in the 3-session HIV education study also evidenced a significant increase in the proportion of participants having safe sex with regular partners. In the trials without HIV education, no change in safe sex practices was found, and change in condom use was observed only among female participants. *Conclusions.* These findings are consistent with recommendations that HIV education/counseling should be provided to individuals in substance abuse treatment. A randomized controlled trial to confirm these results may be warranted. This trial is registered with NCT00033033, NCT00086255, NCT00015106, and NCT00015132.

## 1. Introduction

Approximately 47,800 Americans contract HIV annually, with sexual contact accounting for about 87% of transmissions [1]. Multiple studies have established an association between crack/cocaine use and increased sexual risk behavior (SRB) [2–4]. While drug abuse treatment is, in and of itself, HIV prevention [5], a comprehensive approach includes HIV education/counseling to help patients reduce risky behaviors [6]. However, a 2011 survey of substance treatment programs in the United States found that 43% do not provide HIV education/counseling [7]. Research on the efficacy of HIV education for reducing SRB in crack/cocaine users has been primarily limited to out-of-treatment individuals [8–10]. To our knowledge, two studies have evaluated the efficacy of HIV education for cocaine dependent individuals in treatment and they focused on the ability of the intervention to increase HIV/AIDS knowledge and not on reducing SRB [11, 12]. Substance treatment programs have limited resources and, thus, the HIV education offered typically needs to be simple and to require minimal or no supervision. The question is whether such HIV education would be effective in reducing HIV risk behaviors in a way that is clinically meaningful. The present paper provides preliminary data for answering this question. Specifically, we present data from two cocaine-dependence clinical trials in which HIV education was provided in addition to cognitive behavioral therapy (CBT) for cocaine dependence. In these before and after evaluations of the efficacy of HIV education and substance abuse treatment for increasing safe sex, we selected measures with the greatest public health significance: total abstinence or consistent condom use [13]. We also present, for comparison purposes, a pooled analysis of two cocaine dependence clinical trials which offered only CBT, without HIV education.

## 2. Method

### 2.1. Participants and Procedures

To investigate before and after changes in SRB, this paper presents data from two placebo-controlled randomized multisite cocaine dependence trials, one evaluating reserpine [14] and one evaluating tiagabine [15]. The participants and procedures for these trials are described in detail elsewhere [14, 15].

### 2.2. Study Treatments

In the reserpine and tiagabine trials, a master's level clinician provided each participant with an hour of individual CBT on a weekly basis for 12 weeks. Both studies included HIV education, covering the topics outlined in the “General Guidelines” section of Table 1, with each site providing education in accordance with their state regulations. A sample curriculum, utilized by two sites in the tiagabine study, is provided in Table 1. In the reserpine trial, participants were offered a single session of HIV education. In the tiagabine trial, participants were scheduled for three HIV education sessions, with sessions at baseline, study week 12, and study week 19. Since evaluating the efficacy of HIVeducation in reducing SRB was a secondary goal of the studies, the resources for systematic clinical monitoring and supervision of these sessions were not available; although this is a methodological weakness of the study design, this lack of standardized monitoring/supervision is consistent with the manner in which HIV education will be provided in most substance treatment programs.

### 2.3. Measures

In the reserpine and tiagabine trials, SRB was assessed with the interviewer-administered HIV-risk-taking behavior scale (HRBS) [16], which assesses sexual practices with different types of partners (i.e., regular, casual, and customers). All items were completed based on behavior during the prior month. The good reliability and validity of the HRBS have been established [16, 17]. In the tiagabine trial, the HRBS was completed three times: (1) at baseline, prior to the first HIV education session, (2) at week 12 following the second HIV education session, and (3) at week 19 following the third HIV education session. In the reserpine trial, the HRBS was completed twice, once at baseline and once at study week 12. In both trials, the HRBS was administered by a research assistant. To evaluate the efficacy of HIV education for increasing safe sex, measured as either total abstinence or consistent condom use, we recoded the HRBS items assessing the frequency of condom use as 0 for participants who were abstinent or who used condoms every time when having sex and 1 for participants reporting inconsistent condom use.

### 2.4. Trials without HIV Education

To investigate whether changes in SRB might occur in a clinical trial setting in the absence of HIV education, we analyzed data from two earlier pharmacotherapy cocaine dependence trials in which CBT, but no HIV education, was provided by a master's level therapist. These trials were both ten-week outpatient studies conducted using the Cocaine Rapid Efficacy and Safety Trial (CREST) study design in which three medications and an unmatched placebo are evaluated. The first trial evaluated reserpine, gabapentin, and lamotrigine [18] while the second evaluated tiagabine, sertraline, and donepezil [19]. Since these trials were relatively small (*n* = 60 and *n* = 67, resp.) and were conducted by the same investigators and in the same geographic region, these datasets were combined for the present analysis.

The participants and procedures for these trials are described in detail elsewhere [18, 19]. In both CREST trials, SRB was evaluated at baseline and study week 8 using the Risk Assessment Battery (RAB) [20]. To evaluate safe sex, measured as either total abstinence or consistent condom use, we recoded the RAB item assessing the frequency of condom use as 0 for participants who were abstinent or who used condoms every time when having sex and 1 for participants reporting inconsistent condom use. Unlike the HRBS, which assesses sexual practices for different partner types, the RAB assesses sexual practices without regard to partner type and, thus, yielded one measure of safe sex.

### 2.5. Data Analysis

Participants were included in the data analysis if they completed all of the scheduled SRB assessments (i.e., 3 assessments in tiagabine; 2 in reserpine; 2 in the CREST trials). The rationale for including only completers was that we wanted to include only the participants who received all 3 of the HIV education sessions in the tiagabine study. Since the reserpine and tiagabine trials included different assessment time frames, these datasets were analyzed separately. Within each dataset, analysis was completed for two sets of participants: one including all participants who completed the scheduled SRB assessments and one including only participants reporting sexual nonabstinence during the 30 days assessed. The rationale for conducting the analyses for the subset reporting sexual nonabstinence was to determine whether any decrease in the proportion of participants having unsafe sex was due solely to participants being abstinent during a given 30-day period or to individuals who were using condoms consistently during sex.

We utilized GENMOD (SAS Institute Inc.) for all analyses. The first step in each analysis was to determine whether there were any significant effects for study site, medication condition, or gender and thus a GEE analysis was conducted including each of these variables. These analyses revealed a significant site effect for the reserpine analyses for regular partners and, thus, site was included as a fixed effect. Otherwise, the GEE analyses regressed the outcome measures only against time. For the tiagabine data, any analysis with a significant time effect was followed up by a GEE analysis treating time as a class variable and including contrasts (e.g., baseline versus week 12, etc.) to determine the source of the significance.

## 3. Results

### 3.1. Sample Characteristics

Of the 141 tiagabine participants, 65 (46%) completed the three HRBS assessments. Of the 119 reserpine participants, 83 (69%) completed the two HRBS assessments. The 65 tiagabine participants are referred to as the substance use disorder treatment plus 3-session HIV group (SUD-3-HIV), while the 83 reserpine participants are referred to as the substance use disorder treatment plus 1-session HIV group (SUD-1-HIV).  Table 2 provides demographic and baseline characteristics for four groups: (1) tiagabine participants who completed the three HRBS assessments (i.e., the SUD-3-HIV group), (2) tiagabine participants who did not complete the three HRBS assessments, (3) reserpine participants who completed the two HRBS assessments (i.e., the SUD-1-HIV group), and (4) reserpine participants who did not complete the two HRBS assessments. Comparisons evaluated the existence of significant baseline differences between the groups. The Chi-square analyses were conducted for the categorical variables, while independent *t*-tests were conducted for continuous variables.

The comparisons of the tiagabine completers and noncompleters revealed no significant differences. The comparisons of the reserpine completers and noncompleters revealed a significant difference in race (*χ* ^2^ = 4.84, *P* < 0.05), with significantly more African Americans in the reserpine completers. SUD-3-HIV and SUD-1-HIV group comparisons revealed several significant differences including a greater proportion of SUD-1-HIV participants being employed (*χ* ^2^ = 18.95, *P* < 0.01). In addition, a greater proportion of the SUD-3-HIV participants engaged in unsafe sex (*χ* ^2^ = 3.88, *P* < 0.05) and inconsistent condom use (*χ* ^2^ = 4.22, *P* < 0.05) with their regular partners at baseline. Finally, a greater proportion of the SUD-3-HIV participants, compared to the SUD-1-HIV participants, used condoms inconsistently with customers at baseline (*χ* ^2^ = 5.00, *P* < 0.05).

### 3.2. Unsafe Sex

**Figure 1:**
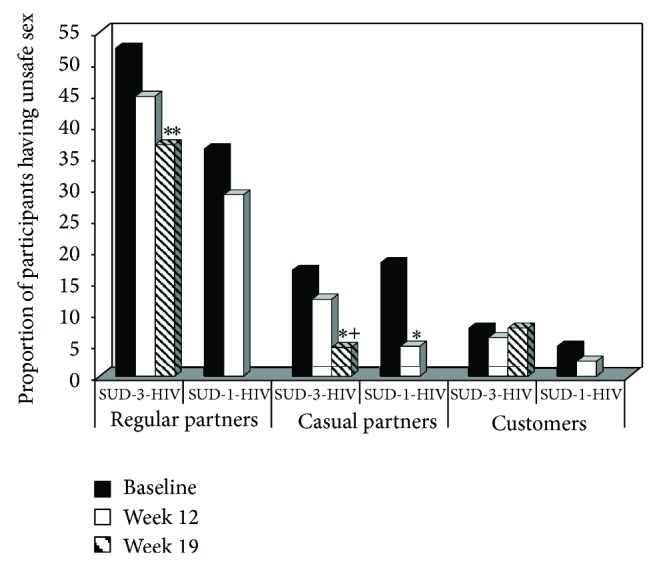
Proportion of participants having unsafe sex as a function of sexual partner type, treatment group, and time. Participants completed either a trial in which they received substance use disorder treatment plus a 3-session HIV education intervention (SUD-3-HIV) or a trial in which they received substance use disorder treatment plus a 1-session HIV education intervention (SUD-1-HIV). The solid black bars represent baseline, the solid white bars represent study week 12, which was the last week for the SUD-1-HIV group, and the striped bars represent study week 19, which was the last week for the SUD-3-HIV group. ^**^ *P* < 0.01 compared to baseline; ^*^ *P* < 0.05 compared to baseline; ^+^ *P* < 0.05 compared to week 12.

The unsafe sex analyses included all of the SUD-3-HIV or SUD-1-HIV participants, regardless of their sexual activity.  Figure 1 displays the proportion of SUD-3-HIV and SUD-1-HIV participants engaging in unsafe sex as a function of time and partner type. The SUD-3-HIV group evidenced significant decreases in the proportion of participants having unsafe sex with regular partners as a function of time (*Z* = 2.77, *P* < 0.01), an effect not seen for the SUD-1-HIV participants (*Z* = 0.93, *P* > 0.05). The analyses of unsafe sex with casual sexual partners revealed a significant decrease in the proportion of participants having unsafe sex for both the SUD-3-HIV (*Z* = 2.37, *P* < 0.05) and SUD-1-HIV (*Z* = 2.47, *P* < 0.05) groups as a function of time. The analyses of unsafe sex with customers revealed no significant time effect for either the SUD-3-HIV (*Z* = 0.01, *P* > 0.05) or SUD-1-HIV (*Z* = 0.82, *P* > 0.05) groups.

### 3.3. Inconsistent Condom Use

**Figure 2:**
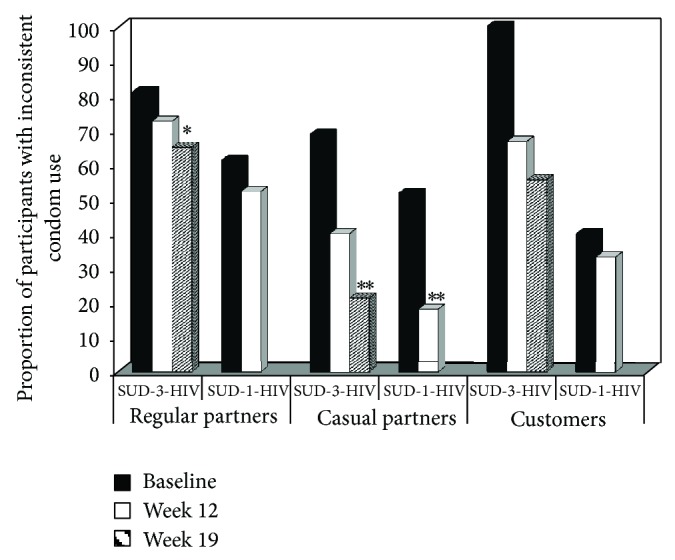
Proportion of participants reporting inconsistent condom use as a function of sexual partner type, treatment group, and time. Participants completed either a trial in which they received substance use disorder treatment plus a 3-session HIV education intervention (SUD-3-HIV) or a trial in which they received substance use disorder treatment plus a 1-session HIV education intervention (SUD-1-HIV). The solid black bars represent baseline, the solid white bars represent study week 12, which was the last week for the SUD-1-HIV group, and the striped bars represent study week 19, which was the last week for the SUD-3-HIV group. ^***^ *P* < 0.01 compared to baseline; ^*^ *P* < 0.05 compared to baseline.

Analyses of inconsistent condom use included only participants reporting sexual nonabstinence during the 30 days assessed.  Figure 2 displays the proportion of SUD-3-HIV and SUD-1-HIV participants using condoms inconsistently as a function of time and partner type. The SUD-3-HIV group evidenced significant decreases in the proportion of the participants using condoms inconsistently with regular partners as a function of time (*Z* = 2.77, *P* < 0.01), an effect not seen for the SUD-1-HIV participants (*Z* = 0.89, *P* > 0.05). The analyses for casual sexual partners revealed a significant decrease in the proportion of the participants using condoms inconsistently for both the SUD-3-HIV (*Z* = 2.71, *P* < 0.01) and SUD-1-HIV (*Z* = 2.64, *P* < 0.01) participants as a function of time. The analyses for customers revealed no significant time effect for either the SUD-3-HIV (*Z* = 1.74, *P* = 0.083) or SUD-1-HIV (*Z* = 0.12, *P* > 0.05) participants; this lack of significance is likely due to the very small sample sizes for this comparison, with the sample being as small as five in some cells.

### 3.4. CBT for Cocaine Dependence without HIV Education Comparison

The results described above suggest that there were significant increases in safe sex behavior in the tiagabine and reserpine trials, but whether this was due to the provision of HIV education is unclear. To help address this question, we completed an analysis following the plan outlined in Section 2.5.

Of the 127 CREST participants, 91 (72%) completed the two RAB assessments and were included in the analysis. The baseline and demographic characteristics of the CREST participants were compared with those of the SUD-1-HIV and SUD-3-HIV participants. The CREST participants were, on average, younger (*X* = 39.4, SD = 6.4) than both the SUD-1-HIV (*t* = −2.06, *P* < 0.05) and SUD-3-HIV (*t* = −3.12, *P* < 0.05) participants and had less education (*X* = 12.4 years, SD = 1.8) compared to the SUD-1-HIV participants (*t* = −2.03, *P* < 0.05). The CREST participants were more likely to be employed (*χ* ^2^ = 42.95, df = 1, *P* < 0.01) and had a larger proportion of minority participants (*χ* ^2^ = 42.95,  df = 1, *P* < 0.01) compared to the SUD-3-HIV participants. All other comparisons were nonsignificant.

The unsafe sex analysis revealed no significant change as a function of time (*Z* = −0.97, *P* > 0.05). The analysis of consistent condom use revealed a significant gender by time interaction effect (*Z* = −2.73, *P* < 0.01); a review of the graph (data not shown) revealed that female participants evidenced decreases in the proportion of participants using condoms inconsistently, an effect not seen for the male participants.

## 4. Discussion

The relationship between crack/cocaine use and sexual risk behavior is well established, but there has been little research on the efficacy of HIV education in reducing this risk in individuals receiving substance abuse treatment. This paper presents the findings from two separate clinical trials using a before and after design in which cocaine dependent individuals were provided with substance abuse treatment and HIV education. In one trial, the participants were offered a single session of HIV education (SUD-1-HIV), while in the other they were offered three sessions of HIV education (SUD-3-HIV). The results suggest that participants in both trials evidenced significant and clinically meaningful increases in safe sex practices. These findings are consistent with recommendations that HIV education/counseling should be provided to individuals in substance abuse treatment.

There are several limitations to the present findings. First, the analyses were conducted post hoc and, thus, should ideally be replicated in a future study in which the analyses are defined a priori. Another potential weakness was the use of self-reported safe sex practices. However, research has found that self-reported SRB correlates highly with corroborator-reported SRB [21]. The lack of systematic clinical monitoring and supervision of the HIV session(s) is another weakness although this lack of standardized monitoring/supervision is consistent with the manner in which HIV education would be provided in most substance treatment programs.

Another potential limitation is the inclusion of participants who completed either a 12-week trial (SUD-1-HIV) or a 19-week trial (SUD-3-HIV). While the lack of baseline differences between the completers and noncompleters suggest that the completers were representative of the entire sample, it would have been ideal to include a greater proportion of participants. The lack of a no HIV education control group is another limitation in that the observed reductions in SRB might be due solely to the effects of the substance abuse treatment provided or to other non-treatment related factors. To help address these two issues, we analyzed data from two cocaine dependence trials in which CBT was provided without HIV education. If the improvements in safe sex behavior observed in the SUD-1-HIV and SUD-3-HIV studies were due solely to the inclusion of study completers or CBT, then one would expect to find similar results from the trials that provided CBT without HIV education. The results indicated no change in safe sex practices for the sample as a whole and improved condom use in only the female participants; these finding are consistent with the idea that HIV education is important for increasing safe sex practices.

The primary strength of the present evaluation is that it is, to our knowledge, the first to evaluate the efficacy of HIV education in reducing SRB in cocaine dependent participants receiving substance abuse treatment. Both trials found significant decreases in the proportion of participants having unsafe sex and using condoms inconsistently with casual partners. The SUD-3-HIV group, which, compared to the SUD-1-HIV group, had a smaller proportion of participants engaging in safe sex practices with regular partners at baseline, also yielded significant increases in the proportion of participants practicing safe sex and using condoms consistently with regular partners. Previous research with substance abusing populations suggests that increasing safe sex practices with regular partners is significantly more challenging than increasing safe sex practices with casual partners [22] and so the present findings are of interest.

In conclusion, the present findings suggest that crack/cocaine addicted individuals receiving substance abuse treatment and HIV education evidenced significant increases on the clinically meaningful measures of safe sex and consistent condom use. It should be noted that even the more intensive 3-session HIV education intervention required less than 1.5 hours of counselor time and, thus, this should be a feasible intervention for substance treatment programs. Given the potential public health significance of these findings, a randomized controlled trial evaluating the effect of HIV education on SRB in crack/cocaine addicted individuals in substance abuse treatment may be warranted.

## Figures and Tables

**Table 1:** General guidelines and a sample curriculum for HIV education.

General guidelines	Sample curriculum
Session 1 (Scr/BL):	Session 1 (Scr/BL, 20–30 min):
(i) Education:	(i) Assess personal HIV risk factors
Modes of transmission	(ii) Education:
High risk behaviors	Brochure: “HIV and AIDS: are you at risk?*” *
Prevention behaviors	Review and Discuss:
Stop drug use	Modes of transmission
Do not share needles	High risk behaviors
Clean “works” before using	Prevention behaviors
Use of condoms	Stop drug use
Use of alcohol swipes	Do not share needles
Use of bleach kits	Clean “works” before using
(ii) HIV testing information:	Use of condoms
What test is for	Demonstrate use of bleach kits
Confidential versus anonymous	handout: “Cleaning Your Works”
Optional	Demonstrate use of alcohol swipes
What +/− test results mean	(iii) Develop personal risk reduction plan:
Anxiety related to waiting for results	Exercise: “Personal Risk Reduction Strategies*” *
(iii) Subject wishes to be tested?	(iv) HIV Pretest Counseling
If yes, talk through the consent	Present HIV testing information:
Obtain signature	Test name, meaning, sensitivity, and specificity
(iv) Offer outside referrals	What test is looking for
	How test will be performed
	What +/− results mean
	Confidential versus anonymous
	Other confidentiality issues
	Where test results will be filed
	Optional, will not affect study participation
	Discuss potential impact of test results
	Handling anxiety related to waiting for results
	How results might affect the participant
	To whom the participant might tell the results
	Worries related to the potential results
	Offer test
	If yes, consent and arrange for test
	If no, provide list of local testing options
	Note: Posttest counseling to occur per local standards at a later date

Session 2 (end TX phase):	Session 2 (End TX Phase, 13–25 min):
(i) No guidelines provided	(i) Assess risk reduction behavior changes
	Identify new strategies employed since session 1
	Retrain strategies if needed
	(ii) Assess continuing high risk behaviors
	(iii) Develop new plan for reducing 1 continuing high risk behavior
	Provide new plan to participant on index card

Session 3 (follow-up):	Session 3 (follow-up, 15–30 min):
(i) No guidelines provided	(i) Assess risk reduction behavior changes
	Identify new strategies employed since session 1
	Retrain strategies if needed
	(ii) Assess continuing high risk behaviors
	(iii) Wrap up
	Review positive risk reduction behavior changes
	Review 1–3 continuing high risk behaviors
	(iv) Provide written list of local HIV resources

**Table 2:** Demographic and baseline characteristics.

	SUD-3-HIV Completers (*N* = 65)	SUD-3-HIV Noncompleters (*N* = 75)	SUD-1-HIV Completers (*N* = 83)	SUD-1-HIV Noncompleters (*N* = 36)
Age (years)	43.3 (7.2)	41.8 (8.1)	41.6 (8.2)	39.6 (6.1)
Sex (% male)	71	69	73.5	64
Race (%)				
African American	71	61	80	64
Caucasian	22	36	13	31
Hispanic	3	3	1	0
Native American/Alaskan	2	0	5	0
Other	2	0	1	5
Administration route (%)				
Smoked	94	96	98	100
Intravenous	1	0	0	0
Intranasal	5	3	2	0
Oral	0	1	0	0
Marital status (%)				
Married	22	16	17	20
Cohabitating	9	5	1	3
Never married	40	32	44	46
Separated/divorced	28	44	36	28
Widowed	1	3	2	3
Education (Years)	12.7 (2.3)	12.7 (2.3)	13.1 (2.0)	12.7 (2.2)
Employment (%)				
Full time	31	27	65	61
Part time	22	23	19	19
Retired/disabled	6	6	5	0
Unemployed	40	44	10	14
Other	1	0	1	6
Cocaine use/last 30	16.3 (9.3)	17.8 (9.5)	18.4 (8.6)	19.0 (8.1)
HRBS Sex Risk Score	4.7 (4.5)	5.1 (3.8)	4.1 (4.1)	4.2 (4.6)
Unsafe sex (%)				
Regular partner	52.3	56.0	36.2	38.9
Casual partner	16.9	16.0	18	22.2
Customer	7.7	6.7	4.8	11.1
Inconsistent condom (%)				
Regular partner	81	75	61.2	73.7
Casual partner	68.8	41.4	51.7	72.7
Customer	100	62.5	40	80

Note. Where not specifically indicated, numbers represent means (standard deviations).
